# Proteomic analysis of non-small cell lung cancer tissue interstitial fluids

**DOI:** 10.1186/1477-7819-11-173

**Published:** 2013-08-05

**Authors:** Shaomin Li, Rui Wang, Mingxin Zhang, Lina Wang, Shaoli Cheng

**Affiliations:** 1 Department of Thoracic Surgery, Second Affiliated Hospital, Medical School, Xi’an Jiaotong University, Xi’an, Shaanxi Province 710004, China; 2 Department of Respiratory Medicine, Xi’an Central Hospital, Xi’an, Shaanxi Province 710003, China; 3 Department of Gastroenterology, Tangdu Hospital, Fourth Military Medical University, Xi’an, Shaanxi Province 710038, China; 4 Department of Emergency, Second Affiliated Hospital, Medical School, Xi’an Jiaotong University, Xi’an, Shaanxi Province 710004, China; 5 Morphological Experiment Centre, Medicine School, Xi’an Jiaotong University, Xia’an, Shaanxi Province 710061, China

**Keywords:** Proteomic, Non-small cell lung cancer, Tissue interstitial fluid, Peroxiredoxin 1

## Abstract

**Background:**

Non-small cell lung cancer (NSCLC) accounts for more than 80% of all lung cancers, and reliable biomarkers are desirable. The present investigation assesses our ability to identify tumor relevant proteins from NSCLC tissue interstitial fluid (TIF).

**Methods:**

Paired TIF was collected from three NSCLC patients at the time of surgery, and resolved by two-dimensional gel electrophoresis and in-gel digestion for proteomic analysis. Differentially expressed spots were extracted from the two-dimensional gel and characterized by high-performance liquid chromatography-tandem mass spectrometry. Then, ELISA was used to verify the expression of peroxiredoxin 1 (PRDX1) in TIF of patients with NSCLC and benign lung disease. Finally, the relationship between expression of PRDX1 and clinicopathological features was determined.

**Results:**

Comparative proteomic analysis showed 24 protein spots were differentially expressed with significant changes, including 11 upregulated proteins and 13 downregulated proteins. Of these, PRDX1 was selected for validation in TIF by Western blot and expression of PRDX1 was confirmed to be upregulated in tumor TIF. It was also demonstrated that PRDX1 was significantly elevated in 40 NSCLC patients with a mean level of 36.0 ng/mL compared to 6.26 ng/mL from 20 patients with benign lung disease. A significant correlation was found between the high level of PRDX1 expression and lymph node metastasis and tumor differentiation.

**Conclusions:**

PRDX1 might be correlated with lymph node metastasis and differentiation, and its elevated expression in TIF may be an adverse biomarker for patients with NSCLC. PRDX1 may be attributed to the malignant transformation of NSCLC, and attention should be paid to a possible target for therapy.

## Background

Lung cancer is the most common cause of cancer mortality in the world, and its incidence is steadily increasing [1]. Non-small cell lung cancer (NSCLC) accounts for more than 80% of all lung cancers. Most NSCLC patients are diagnosed at an advanced stage, and a variety of comprehensive treatments failed to significantly prolong the survival time [2]. Arising from the observation that early-stage disease is amenable to and may be cured by surgical resection, early detection of NSCLC would significantly improve patient clinical outcome [3]. However, there are currently no biomarkers to enable reliable screening for NSCLC.

Currently, the most accurate diagnostic test is a histological examination of a tissue biopsy. A less invasive test from a readily available biofluid such as serum would be greatly beneficial. Unfortunately, serum is an extremely complex biofluid containing high-abundant proteins and differential quantification of potential biomarkers. In particular, potential biomarkers are likely to be present at relatively low abundance for early stage malignancies [4]. Tissue interstitial fluid (TIF) is another sample that has gained increasing attention as a substrate from which candidate protein biomarker discovery can be made. The production of TIF results from build-up of fluid derived from cell secretions due to a lack of a vascular and lymphatic system within the tumor [5]. Because this fluid is directly associated with the tumor, it may be a rich source of tumor-specific biomarkers. Despite its potential value as a source of tumor biomarkers, representative catalogs of TIF proteomes of NSCLC are lacking.

In the current study, TIF was collected from patients diagnosed with NSCLC with matched non-tumor adjacent tissue samples from operation. These TIF samples were analyzed by liquid chromatographytandem mass spectrometry (LC-MS/MS). Several proteins were identified as significantly elevated in abundance in tumor TIF, including peroxiredoxin 1 (PRDX1). The relative abundance of PRDX1 was verified in TIF by Western blot analysis. Furthermore, PRDX1 was found to be elevated in the TIF of patients diagnosed with NSCLC compared to that in benign lung disease, as determined by ELISA.

## Methods

### Patients

NSCLC patients who were confirmed by pathology were collected in the Second Affiliated Hospital of Xi’an Jiaotong University from 2009 to 2010, and also received surgical treatment. Histological classification and differentiation grade were conducted according to 1999 WHO histological classification standards of lung cancer; staging was carried out according to newly revised TNM staging criteria of the International Union against Cancer in 2009. The Institutional Ethics Committee approval for this project was obtained from Second Affiliated Hospital, Medical School, Xi’an Jiaotong University. All the tumor samples used contained more than 50% tumor cells, and were stored at −80°C before use. A total of 40 paired samples were collected, and three tumor samples and three non-tumor adjacent samples were randomly chosen from the tissue archive, and pooled separately. For ELISA assay, samples of 20 patients with benign lung disease were also collected.

### Tissue interstitial fluid collection and processing

The TIF collection was carried out as described previously [6]. Briefly, within 30 minutesof resection, approximately 250 mg of tumor and non-tumor adjacent tissue (NAT) from NSCLC patients was dissected into small pieces (1 to 3 mm^3^), placed into a 6-well plate containing 5 mL PBS and washed for 5 minutes. Tissues were transferred to a microcentrifuge tube containing 0.8 mL fresh PBS and incubated for 1 hour at 37°C to harvest the TIF. The TIF was transferred to a new microcentrifuge tube, centrifuged at 1000 rpm for 2 minutes at 4°C and supernatant was collected. Samples were further centrifuged at 5,000 rpm for 20 minutes.

Samples were then subjected to immunodepletion using the Multiple Affinity Removal System (Hu-14, Agilent Technologies, Inc., Santa Clara, CA, USA) according to the manufacturer’s protocol. Proteins were extracted from immunodepleted TIF using a buffer consisting of 7 M urea, 2 M thiourea, 4% CHAPS, 2% NP-40, 2% pharmalyte, 5 mM PMSF, 1% Triton X-100, 100 mM DTT, and 0.5 mM EDTA, and centrifuged for 1 hour at 4,000 rpm. The supernatant was precipitated with acetone and dried. Protein concentrations were determined using the Bradford assay. All samples were stored at −80°C prior to electrophoresis.

### Two-dimensional gel electrophoresis

First-dimension isoelectric focusing was performed at 20°C for 30,000 VhT using IPGphor^TM^ Isoelectric Focusing Unit (Amersham Pharmacia Biotech AB, Uppsala, Sweden). The second-dimension gel electrophoresis was performed using 12.5% gradient SDS polyacrylamide gels with buffer running solutions containing 1.5 M Tris and 10% SDS. Gels were stained with 0.2% (w/v) silver nitrate [6]. ScanMaker8700 (Amersham Pharmacia Biotech AB) was used for scanning of gels and image analysis software (Image Master 2D Platinum Software 5.0, Geneva Bioinformatics, Geneva, Switzerland) was used to construct average gels from three independent experiments and a comparison between those average gels was performed.

### High-performance liquid chromatography-tandem mass spectrometry

After digestion of interested differently expressed spots, an HPLC system (Surveyor, ThermoFinnigan, San Jose, CA, USA) was applied. Peptides were eluted by using a gradient from buffer A (0.1% formic acid) to buffer B (90% vol/vol acetonitrile, 0.1% formic acid). The HPLC column eluent was eluted directly into the electrospray ionization source of a LCQ-Deca ion trap mass spectrometer (ThermoFinnigan). Automated peak recognition, dynamic exclusion, and daughter ion scanning of the top two most intense ions were performed using the Xcalibur software (ThermoFinnigan, San Jose, CA, USA) [7]. Spectra were scanned over the range 400 to 2,000 mass units. MS/MS data were analyzed using SEQUEST database a computer program that allows the correlation of experimental data with theoretical spectra generated from known protein sequences [8]. All matched peptides were confirmed by visual examination of the spectra, and all spectra were searched against the latest version of the public non-redundant protein database of the NCBI (http://www.ncbi.nlm.nih.gov) or SWISS-PROT (http://web.expasy.org/docs/swiss-prot_guideline.html).

### Western blot

Total protein from each sample (10 mg) was separated on 12% polyacrylamide gel and then transferred electrophoretically onto polyvinylidene difluoride membranes. After blocking for 1 hour with blocking buffer (1 × PBS, 0.5% Tween-20 with 5% non-fat dry milk), the membranes were incubated overnight at 4°C with antibodies against PRDX1 (Abcam, Cambridge, MA, USA; 1:500) The primary antibodies were detected by use of horseradish peroxidase-conjugated secondary antibodies (goat anti-rabbit or mouse immunoglobulin G; Santa Cruz Biotechnology, CA, USA). Visualization of the immunoreactive proteins was accomplished by use of ECL Plus reagents (Amersham Biosciences, Little Chalfont, UK) and exposed to X-ray film. β-actin was used as a loading control.

### ELISA

The levels of abundance of PRDX1 were measured using the peroxiredoxin 1 Human ELISA kit (BioVendor, Candler, NC, USA) according to the manufacturer’s instructions. TIF samples from patients with NSCLC and benign lung pathology were diluted 1:4 prior to measurement and were assayed simultaneously in duplicate. Serial dilutions of PRDX1 standard were assayed in parallel with TIF samples. The optical density was plotted against standard PRDX1 concentrations to generate the standard curve according to the manufacturer’s protocol. A non-parametric Wilcoxon rank-sum test was used to determine the significance of the difference between the median PDRX1 quantities in TIF of patients with benign lung pathology versus patients with NSCLC.

### Statistical analysis

To avoid including in the data analysis proteins with no real change in expression, we selected only spots with a fold-change higher than 2 to be considered for statistical analysis. The student’s *t*-test was used to compare the western blot and ELISA results. To investigate the association with clinicopathologic features, PDRX1 expression values were dichotomized into low and high groups using the median expression value within the cohort as a cutoff. A Fisher’s exact text was used to analyze the relationship between expression levels of PDRX1 and various clinicopathologic characteristics. The difference was considered statistically significant if the *P*-value was less than 0.05.

## Results

### Two-dimensional gel electrophoresis and the analysis of gel images

**Figure 1:**
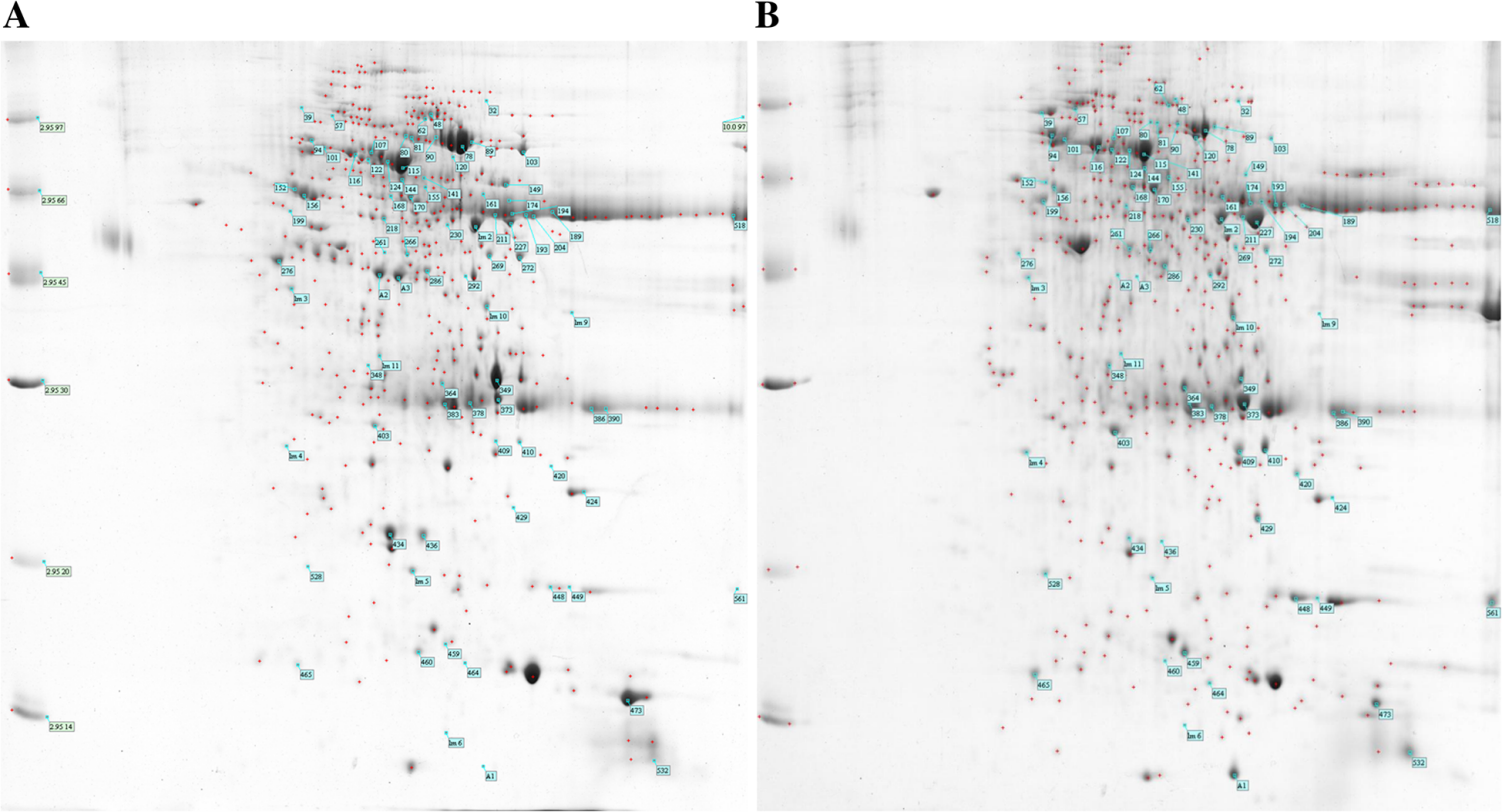
**Differentially expressed proteins in tissue interstitial fluid of non-small cell lung cancer biopsies.** The two-dimensional electrophoresis gel is representative of all the tissue interstitial fluid samples, since it has been obtained by pooling tissue interstitial fluid of all patients. Spot number reflects the number listed for the specific protein in Table 1. Molecular weight (kDa) and isoelectric values (pI) are shown on the image. **(A)** Non-tumor adjacent tissue; **(B)** tumor.

In order to identify proteins associated with NSCLC development, we compared two-dimensional gel patterns of tumor and NAT TIF proteomes. NAT samples were obtained from more than 5 cm away from the tumor margin of the NSCLC. The samples were immunodepleted of serum albumin and immunoglobulin G for minimization of sample bias. The resulting images were analyzed using PDQUEST software (Bio-Rad, Hercules, CA, USA). Typical two-dimensional gel electrophoresis proteome spot patterns of tumor TIF and NAT TIF are shown in Figure 1. Under the same experimental conditions, six gels(three for tumor TIF and three for NAT TIF) were analyzed. In the two-dimensional gel electrophoresis maps of tumor TIF and NAT TIF, 24 protein spots were found to be differentially expressed with changes in the stain density of two-fold or more, including 11 upregulated proteins and 13 downregulated proteins (*P* < 0.05).

### High-performance liquid chromatography-tandem mass spectrometry

**Figure 2:**
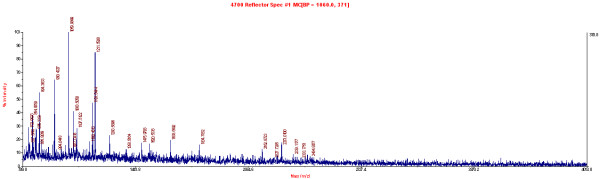
**An example tandem mass spectrum of a peptide.** This spectrum represents one example of a peptide identified by high-performance liquid chromatography mass spectrometry.

**Figure 3:**
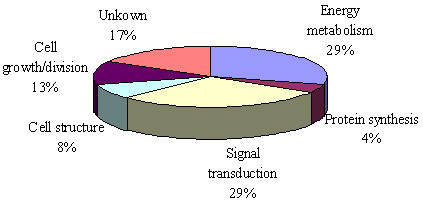
**A comparison of protein identification results.** Detected proteins were mainly involved in energy metabolism and signal transduction.

Twenty four differentially expressed spots were identified by HPLC-MS analysis. All identified different proteins are shown in Table 1. An example (No.409 as shown in Table 1 and Figure 1) of the tandem mass spectrum of the 24 peptides is shown in Figure 2. The identified proteins were next categorized into possible functions according to the classification systems [9]. Detected proteins were mainly involved in energy metabolism and signal transduction (Figure 3). Among those proteins identified, PRDX1 was identified in greater abundance (an average of 15-fold greater spectral count) in NSCLC TIF samples as compared to NAT TIF. We therefore chose PRDX1 as a candidate protein for the following experiments.

**Table 1:** Identification of proteins differentially expressed in non-small cell lung cancer tissue interstitial fluid

**Number**^**a**^	**Name**	**Sequence coverage**^**b**^**(%)**	**Mascot score**^**c**^	**Molecular weight**	**Change(−fold)**
48	LINE-1 type transposase domain-containing protein 1 (L1TD1)	38	46	98789	Down/13.7
90	Serotransferrin	49	99	77014	Down/3.9
94	Heat shock protein HSP 90-beta	39	50	83212	Up/3.1
103	Complement C3	33	96	187030	Down/6.1
116	Heat shock cognate 71 kDa protein	50	106	70854	Up/4.7
122	Heat shock 70 kDa protein 1A/1B	51	122	70009	Down/8.1
156	Vinculin	23	39	123722	Down/3.1
168	Fibrinogen gamma chain	54	103	51479	Up/3.9
170	Fibrinogen beta chain	45	81	55892	Up/2.4
193	Tubulin-specific chaperone E	34	48	59309	Down/7.7
204	GAS2-like protein 1	37	45	72672	Down/5.7
227	Alpha-enolase	50	68	47139	Up/4.3
230	Zinc finger protein 879	42	49	64536	Up/2.7
272	Putative uncharacterized protein FLJ46541	56	49	18178	Down/2.3
349	Zinc finger protein 658B	52	50	94270	Down/3.4
364	ADP-ribosylation factor-related protein 1	71	44	22599	Up/3.5
386	Zinc finger and SCAN domain-containing protein 10	48	42	80336	Down/2.1
390	Serine/threonine-protein kinase ATR	21	49	301172	Down/2.6
403	Glutathione S-transferase P	56	47	23341	Up/3.0
409	Peroxiredoxin-1	37	54	22096	Up/15.0
434	CAP-Gly domain-containing linker protein 3	34	45	59522	Down/7.0
448	Sterol-4-alpha-carboxylate 3-dehydrogenase, decarboxylating	28	41	41874	Up/6.1
459	t-SNARE domain-containing protein 1	23	34	55914	Up/4.8
473	Hemoglobin subunit alpha	68	56	15248	Down/4.4

^a^ Protein spot numbers are highlighted in Figure 1.

^b^ Sequence coverage of the matched peptides in protein by NCBInr Database.

^c^ MASCOT score is a parameter that correlates with the quality of the search result on the MASCOT and SWISS-PROT. High scores are indicative of successful, valid identifications.

### Confirmation of upregulated expression of peroxiredoxin 1 by Western blot and ELISA

**Figure 4:**
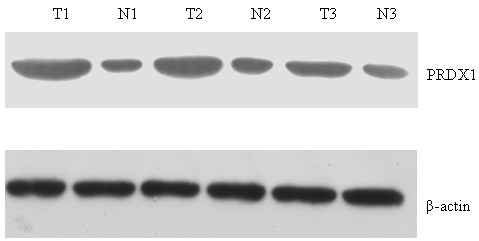
**Western blot analysis of peroxiredoxin 1 in tissue interstitial fluid of tumor and non-tumor adjacent tissue.** Western blot analysis revealed markedly upregulated peroxiredoxin 1 (PRDX1)expression in tumor tissue interstitial fluid. β-actin was used as a loading control.

**Figure 5:**
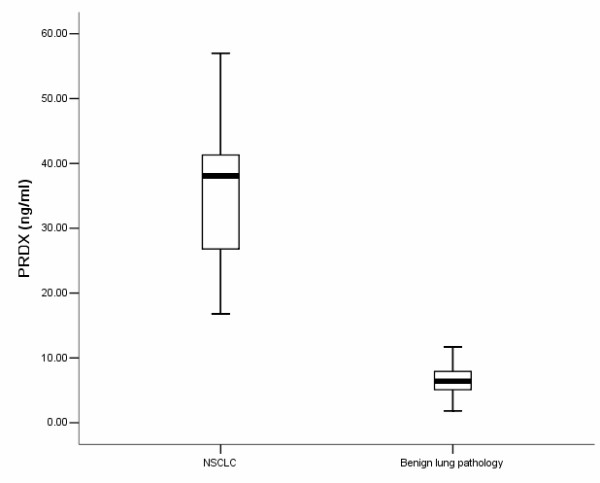
**Peroxiredoxin 1is elevated in tissue interstitial fluid from non-small cell lung cancer patients.** Tissue interstitial fluid from 40 patients diagnosed with non-small cell lung cancer (NSCLC) and tissue interstitial fluid from 20 patients with benign lung pathology was evaluated for peroxiredoxin 1 (PRDX) presence and quantity by ELISA. There was a statistically significant difference between the mean PDRX levels in the two groups.

We first confirmed the expression of PRDX1 in the NSCLC and NAT TIF by western blot. The results showed that PRDX1 was upregulated in NSCLC TIF compared to that of NAT (Figure 4). Then, we applied ELISA to detect the expression of PRDX1 in TIF of samples of NSCLC, NAT and benign lung disease. The mean level of PRDX1 from these 40 NSCLC patients was determined to be 36.0 ± 5.48 ng/mL and 6.26 ± 3.12 ng/mL from the 20 benign lung populations as measured using a commercially available PDRX1 ELISA (Figure 5). Furthermore, expression of PRDX1 was significantly related to lymph node metastasis and differentiation (Table 2).

**Table 2:** Relationship between clinicopathologic variables and the expression status of peroxiredoxin 1

**Variables**	**Total**	**PRDX1**		***P***
		**Low**	**High**	
Age				0.332
< 60	18	4	14	
≥60	22	8	14	
Gender				0.697
Male	31	10	21	
Female	9	2	7	
Smoker status				0.530
Yes	23	6	17	
No	17	6	11	
Histology				0.808
AC	16	4	12	
SCC	23	8	15	
ASC	1	0	1	
T-stage				0.729
T_1-2_	24	8	16	
T_3-4_	16	4	12	
p-TNM stage				0.677
I-II	22	6	16	
III-IV	18	6	12	
Lymph node metastasis				0.006
No	20	10	10	
Yes	20	2	18	
Differentiation				0.004
Poorly differentiated	17	1	16	
Highly differentiated	23	11	12	

*AC*, adenocarcinoma; *ASC*, adenosquamous carcinoma; *PRDX1*, peroxiredoxin 1; *pTNM*, pathological tumor-node-metastasis; *SCC*, squamous cell carcinoma.

## Discussion

Because potential protein biomarkers are more abundant in the tumor tissue microenvironment than serum, an increased focus in proteomics is to utilize proximal fluids. As one example of a proximal fluid, TIF has proven to be an important resource for cancer biomarker discovery). Celis and colleagues [6] investigated proteins from breast cancer and normal TIF, where it was demonstrated that proteins identified by MS-based proteomics could be externally validated in a tissue microarray containing over 70 breast carcinoma tissue samples [10]. Furthermore, there are lots of reports of TIF in renal cell carcinoma, head and neck squamous carcinoma, and ovarian cancer [4,11,12], but there is limited research on TIF in NSCLC.

In our analysis, 24 protein spots were identified between tumor TIF and NAT, including 11 upregulated proteins and 13 downregulated proteins. Some identified proteins function as potential tumor-associated proteins. Alpha-enolase (ENO1), which was found to be over-expressed in our study, has also been reported to be upregulated in several types of cancer [13-15]. Furthermore, ENO1 was identified as a tumor antigen in lung cancer with different proteomic methods by two independent scientific groups [16,17]. Moreover, its overexpression was associated with clinical outcomes in lung cancer [17]. Glutathione S-transferase P (GSTP) is another upregulated protein in our study. Previous studies revealed there is an association between high levels of GSTP and malignant diseases and drug-resistant cancer [18].

As the fold of change of PRDX1 was the most significant, we chose it for confirmation and further experiment. PRDX1 is a member of the redox-regulating protein family, peroxiredoxins [19]. PRDX1 is of particular interest due to its known elevated level of gene expression in numerous cancers [20-22], including NSCLC [23-25]. PRDX1 presence in carcinomas is associated with inhibition of apoptosis, equating to increased tumor survival [20-25]. In addition, several other peroxiredoxin family members (PRDX2-6) are thought to be linked to lung cancer [26-28]. Although much effort has been devoted to the investigation of PRDX1 function in cultured cells, animal systems and tissues, no research has been undertaken to evaluate the value of PRDX1 in TIF in NSCLC.

In the present study, the presence of PRDX1 in TIF from NSCLC patients was verified by Western blot to be elevated in abundance in tumor TIF as compared to NAT TIF. We attempted to determine the levels of PRDX1 in TIF of samples from malignant or benign lung disease using a highly validated, commercially available ELISA. These results demonstrated that patients with NSCLC had a six-fold elevated level of PRDX1 in their TIF compared to patients with benign lung pathology (*P* < 0.05). Furthermore, expression of PRDX1 was significantly related to lymph node metastasis and differentiation. These results suggest that PRDX1, in addition to serving as a biomarker, may also serve as a therapeutic target. Based on the hypothesis that a subset of TIF proteins represents shed/secreted proteins from the microenvironment, the TIF proteins may be exchangeable and detectable within peripheral blood. Thus we will detect the expression and diagnostic value of PRDX1 in blood in a further study.

## Conclusion

In conclusion, we utilized a mass spectrometry based proteomics workflow to identify tumor specific proteins from TIF from NSCLC patients. Among the differentially abundant proteins identified from TIF, PRDX1 was demonstrated to be present in TIF by Western blot and to be elevated in NSCLC patients by ELISA. Our study adds further support to the hypothesis that TIF represents an attractive proximal biofluid for conducting candidate biomarkers of NSCLC.

### Consent

Written informed consent was obtained from the patients for the publication of this report and any accompanying images.

## Abbreviations

ELISA: Enzyme-linked immunosorbent assay; ENO1: Alpha-enolase; GSTP: Glutathione S-transferase P; HPLC: High-performance liquid chromatography; LC-MS/MS: Liquid chromatography tandem mass spectrometry; NAT: Non-tumor adjacent tissue; NSCLC: Non-small cell lung cancer; PBS: Phosphate buffered saline; PRDX1: Peroxiredoxin 1; TIF: Tissue interstitial fluid.

## Competing interests

The authors declare that they have no competing interests.

## Authors’ contributions

SL and RW participated in the design and conducting experiments, data analysis, and final drafting and writing of the manuscript. SL, RW, MZ and LW all contributed to these experiments. SL and SC were closely involved in research design and drafting of the final manuscript. All authors read and approved the final manuscript.
